# Comparison of functional outcomes of off-clamp laparoscopic partial nephrectomy access techniques: A preliminary report

**DOI:** 10.1590/S1677-5538.IBJU.2019.0734

**Published:** 2020-11-18

**Authors:** Mehmet Necmettin Mercimek, Ender Ozden

**Affiliations:** 1 Samsun Liv Hospital Department of Urology IlkadimSamsun Turkey Department of Urology, Samsun Liv Hospital, Ilkadim, Samsun, Turkey; 2 Ondokuz Mayis University Faculty of Medicine Department of Urology AtakumSamsun Turkey Department of Urology, Faculty of Medicine, Ondokuz Mayis University, Atakum, Samsun, Turkey

**Keywords:** Laparoscopy, Nephrectomy, Methods

## Abstract

**Objective:**

This study aims to compare renal functional outcomes of access techniques in patients who underwent off-clamp (Off-C) laparoscopic partial nephrectomy (LPN).

**Materials and Methods:**

Thirty-four Off-C LPNs in patients with functioning contralateral kidney from March 2011 to June 2018 were included in the study. Twenty-two patients underwent transperitoneal, 12 patients underwent retroperitoneal Off-C LPN. The primary outcome was glomerular filtration rate changes over time, postoperatively. The secondary outcome was the evaluation of trifecta and pentafecta rate.

**Results:**

Preoperative demographics, tumor size (26.59 vs. 22.83mm, p=0.790), RENAL score (5.45 vs. 5.33, p=0.990), operation time (79.95 vs. 81.33 min, p=0.157), blood loss (170.23 vs. 150.83mL, p=0.790) were similar in both groups. Although preservation of renal function was better in group 2 in the early period, similar results were found in both groups at the end of the first year, postoperatively. No positive surgical margin and postoperative major complications were detected in any patient. While trifecta goals were achieved in all the patients in the cohort, pentafecta rates were 90.9% and 91.7% in the transperitoneal and retroperitoneal groups, respectively.

**Conclusions:**

Transperitoneal and retroperitoneal access were found to have similar outcomes in terms of preservation of renal function at the end of the first year postoperatively. Off-C LPN may be considered as a safe and effective treatment option in patients having non-complex renal tumors.

## INTRODUCTION

It is generally accepted that partial nephrectomy (PN) is a standard procedure due to having equal oncological and better functional outcomes when compared to radical nephrectomy (RN) in patients with cT1 renal tumors, whenever it is technically feasible (1).

Preoperative baseline renal function (RF), renal parenchyma preserved, and warm ischemia time (WIT) are strongly associated with renal functional recovery after PN. Therefore, minimizing or even eliminating the ischemia time as well as preserving the quantity of remnant renal parenchyma are the crucial modifiable factors that would have a positive effect on renal functional recovery after PN (2).

While a definite cut-off value for the duration of global ischemia that should not be exceeded during PN in humans has not yet to be defined, various techniques including selective (minimal) or off-clamp (Off-C) have been described to reduce the negative effect of global ischemia on RF (2).

In the present study, we aimed to investigate the effect of the surgical approach on functional and oncological outcomes of Off-C LPN. To date, there is no study available to evaluate the impact of transperitoneal vs. retroperitoneal Off-C LPN on both surgical and oncological outcomes.

## MATERIALS AND METHODS

The study has been approved by the Ondokuz Mayıs University Clinical Research Ethics Committee (OMU KAEK 2019/539), and it conforms to the provisions of the Declaration of Helsinki in 1995. All participants provided written informed consent to take part in the study.

From November 2009 to June 2018 a total of 44 Off-C LPNs were performed at a tertiary care university hospital in Turkey. Patients with bilateral renal tumors (n=4), solitary kidney (n=4), and unilateral multiple (≥2) renal tumors (n=2) were excluded. The remaining 34 patients with a contralateral functioning kidney were included in the study. The patients were divided into two groups according to laparoscopic access technique: group 1; transperitoneal LPN (T-LPN), n=22 and group 2; retroperitoneal LPN (R-LPN), n=12.

The clinical diagnosis was determined using radiological imaging methods. Triphasic contrast-enhanced computed tomography (CT) was used to indicate tumor anatomy, and three-dimensional (3D) images were obtained. The complexity of the tumor was evaluated by R.E.N.A.L nephrometry score (3).

The demographic characteristics of patients, including age, sex, body mass index (BMI) and systemic diseases such as diabetes mellitus (DM), hypertension (HT), and coronary artery disease (CAD) as well as the clinical tumor characteristics were recorded.

The final decision of access technique was obtained according to renal vascular anatomy, R.E.N.A.L nephrometry score (RNS), tumor characteristics, and vascular supplies of the tumor as well as surgeon's preference.

### Surgical Technique

In our clinical practice, the patients who underwent LPN routinely hospitalize one day before surgery. Intravenous fluid is given according to BMI. Fluid support is continued until enough oral intake achieved. Then, the importance of daily fluid intake is being reminded to the patients before the discharge. All operations were performed by the same surgeon (EO). Both T-LPN and R-LPN were performed under similar principles. The gas pressure was increased up to 12mmHg to create a retroperitoneal or a transperitoneal space. The renal artery, renal vein and ureter were dissected and then isolated with vascular silicon tapes. The kidney was mobilized from the surrounding tissues as much as possible; attention was paid to the preservation of perirenal fatty tissue adjacent to the tumor. Laparoscopic ultrasound was used to detect the mass and determine the surgical margin. Monopolar hook was used to score the surgical margin. Then, the renal tumors were completely excised by a cold scissors with a thin negative margin. The tumor bed was sutured in two layers, supported with hem-o-lock clips. A great effort was made both to secure the remnant renal parenchyma and prevention of bleeding during tumor bed control. The specimen was extracted in an Endo-bag and a drain was placed in surgical field.

#### Outcome assessment

Perioperative and postoperative findings including surgical technique, operation time (OT), estimated blood loss (EBL), preoperative and postoperative hemoglobin (Hgb) values, length of hospital stay (LOS), final pathology, surgical margin status, and perioperative and postoperative complications were recorded. Serum creatinine levels of preoperative, postoperatively at 1st day, at 1st month, at 6^th^ month and at 1st year were also recorded. Estimated glomerular filtration rate (eGFR) was calculated using the Chronic Kidney Disease Epidemiology Collaboration (CKD-EPI) equation (4). Furthermore, patients were classified according to eGFR values as grade 1-5 stages of the CKD classification (5). Postoperative complications were graded according to the modified Clavien-Dindo classification system (I-V) (6).

Trifecta refers to a short-term assessment of PN outcomes and pentafecta is an evaluation of long-term outcomes of PN. Since neither hilar nor segmental vessels were clamped in any of the cases, all LPN procedures have warm ischemia time of zero. Patients with a negative surgical margin, and absence of postoperative complications (Clavien-Dindo ≥ grade 3) were accepted to achieve the trifecta outcomes. Pentafecta is defined as trifecta criteria plus >90% preservation of eGFR and no stage upgrade of chronic kidney disease from preoperative up to 12 months after LPN.

### Statistical Analysis

Shapiro Wilks test was used for normality of parameters in the evaluation of study data. Besides use of descriptive statistical methods (mean, standard deviation, frequency) in the evaluation of study data; for the comparison of quantitative data, Student's t test was used for comparing parameters with normal distribution between the two groups and Mann-Whitney U test was used for comparing parameters with non-normal distribution between the two groups. Fisher's exact test and chi-square test were used to analyze the correlation between categorical variables. Significance was taken as p<0.05. The data were analyzed using Statistics Package for Social Sciences version 24 (IBM SPSS®, Armonk, NY).

## RESULTS

A total of 34 patients who underwent T-LPN (N=22, 64.7%) and R-LPN (N=12, 35.3%) were retrospectively analyzed. The mean age was 58±14 years (range 29-81), the mean tumor size was 25±13mm (range 10-60), median RNS was 5 (range 4-8), mean follow-up 51.29±25.29 months (range 12-89). Demographic data of patients and clinical characteristics of tumors are demonstrated in Table-1. There were no statistically significant differences between the T-LPN vs. R-LPN groups in terms of demographics and clinical tumor characteristics including age, BMI, HT, DM, CAD, clinical tumor stage, the mean tumor size (p=0.793), tumor laterality (p=0.642), as well as the mean RNS (p=0.990).

**Table 1:** Demographics Features and Clinical Tumor Characteristics.

Variable*		Transperitoneal (n=22)	Retroperitoneal(n=12)	P value
Age (years)		57.27±14.4	60.08±14.9	0.595^a^
**Gender**				**0.236**^b^
	Female	12 (54.5)	4 (33.3)	
	Male	10 (45.5)	8 (66.7)	
BMI (kg/m^2^)		26.71±3.56	26.55±2.46	0.887^a^
**Hypertension**				**0.350**^b^
	Yes	11 (50)	4 (33.3)	
	No	11 (50)	8 (66.7)	
**Diabetes Mellitus**				**0.406**^b^
	Yes	3 (13.6)	3 (25)	
	No	19 (86.4)	9 (75)	
**Coronary artery disease**				**0.676**^b^
	Yes	5 (22.7)	2 (16.7)	
	No	17 (77.3)	10 (83.3)	
**Clinical T stage**				**0.293**^b^
	T1a	17 (77.3)	11 (91.7)	
	T1b	5 (22.7)	1 (8.3)	
Tumor size (mm)		26.59±14.57	22.83±9.29	0.790^c^
**Laterality**				**0.642**^b^
	Right	11 (50)	5 (41.7)	
	Left	11 (50)	7 (58.3)	
RNS		5.45±1.26	5.33±0.89	0.990^c^

a Independent samples t-test,

b Pearson's chi-square test,

c Mann-Whitney U test, BMI - Body mass index, mm - millimeter, RNS - RENAL nephrometry score,

* Continuous variables are presented as mean±SD, categorical variables as number (%)

The perioperative, postoperative and renal functional outcomes are demonstrated in Table-2. When group 1 and group 2 were compared in terms of surgical outcomes, both groups were statistically similar in terms of OT (79.95±25.94 vs. 81.33±41.78 min, p=0.157), EBL (170.23±79.44 vs. 150.83±99.95mL, p=0.790), Hgb drop (1.65±1.12 vs. 1.35±0.65g/dL, p=0.405), LOS (2.77±0.68 vs. 2.42±0.66 day, p=0.155), postoperative complication rate (9.1% vs. 8.3%, p=0.721), and preoperative eGFR values [103.60 (range 52.3-150) vs. 90.28 (range 51.96-155.04), p=0.186]. However, the mean decrease in eGFR on the first postoperative day was statistically different in both groups (12.28±13.30 vs. 2.89±2.99, p=0.04). Furthermore, ΔeGFR (preoperative eGFR- the first year of eGFR) was also found statistically different up to the postoperative first year although the mean eGFR values were similar. At the end of the postoperative first year, the mean ΔeGFR was found to be similar in the T-LPN and R-LPN groups (5.44±6.43 vs. 2.37±3.75, p=0.141).

**Table 2:** Perioperative, Postoperative and Renal Functional Outcomes.

Variable*			Transperitoneal (n=22)	Retroperitoneal (n=12)	P value
OT (min)			79.95±25.94	81.33±41.78	0.157^a^
EBL (mL)			170.23±79.44	150.83±99.95	0.790^a^
Preoperative HGB (g/dL)			13.40±1.90	13.53±1.14	0.833^b^
Postoperative HGB (g/dL)			11.75±1.53	12.18±1.22	0.411^b^
LOS (day)			2.77±0.68	2.42±0.66	0.155^a^
**Final pathology**					**0.860**^c^
	RCC		14 (63.6)	8 (66.7)	
	Benign		8 (36.4)	4 (33.3)	
**eGFR (mL/min/1.73m**^2^)					
Preoperative			103.61±27.38	90.28±27.59	0.186^b^
	**Postoperative**				
		1-day	91.32±19.53	87.38±27.56	0.631^b^
		1-month	96.20±21.81	88.16±27.32	0.355^b^
		6-month	97.46±22.66	88.93±22.66	0.367^b^
		1-year	98.17±23.82	87.91±27.24	0.286^b^
Δ**eGFR (mL/min/1.73m**^2^)					
		1-day	12.28±13.30	2.89±2.99	0.04^a^
		1-month	7.40±7.53	2.11±2.0	0.05^a^
		6-month	6.11±6.88	1.35±1.36	0.04^a^
		1-year	5.44±6.43	2.37±3.73	0.141^b^

a Mann-Whitney U test,

b Independent samples t-test,

c Pearson's chi-square test, EBL - estimated blood loss, eGFR - estimated glomerular filtration rate, HGB - hemoglobin, min - minute, mL - milliliter, LOS - length of hospital stay, OT - operation time,

* Continuous variables are presented as mean±SD, categorical variables as number (%).

Trifecta and pentafecta outcomes are demonstrated in Table-3. None of the cases in both groups had any perioperative complications or need to convert to open surgery. According to the final pathology report, none of the patients in the cohort had a positive surgical margin. When the overall cohort was evaluated in terms of the mean relative change in percentage of ΔeGFR, it was found 7.8% on the first postoperative day, 4.8% on the first month, 3.8% on the sixth month, and 3.9% in the first year, respectively. Trifecta and pentafecta rates were found to be similar in the T-LPN and R-LPN groups. Trifecta was achieved in all patients in both groups. Overall complications were identified in 8.8% of the cohort. In the T-LPN group, two patients with preoperative hemoglobin levels close to the lower limit of the normal values required blood transfusion, postoperatively. In the R-LPN group, one patient had a fever in the early postoperative period. However, all of them were < grade III complications according to the Clavien-Dindo classification system. Preoperative (p=0.074) and first-year (p=0.697) CKD stages were found to be similar. CKD stage increase was identified only in one patient in both groups. At the end of the first year, >90 eGFR preservation was achieved as 20 (90.9%) and 11 (91.7%) in the T-LPN and R-LPN groups, respectively.

**Table 3:** Trifecta and pentafecta outcomes.

Variable*		Transperitoneal (n=22)	Retroperitoneal (n=12)	P value
Negative surgical margin		22 (100)	12 (100)	–
Ischemia time		0	0	–
**Preoperative CKD stages**				**0.774**^a^
	Stage 1	15 (68.2)	7 (58.3)	
	Stage 2	5 (22.7)	3 (25)	
	Stage 3a	2 (9.1)	2 (16.7)	
**1**^st^ **year CKD stages**				**0.69**^7a^
	Stage 1	14 (63.6)	6 (50)	
	Stage 2	6 (27.3)	4 (33.3)	
	Stage 3a	2 (9.1)	2 (16.7)	
**eGFR preservation in the 1**^st^ **year**				**0.721**^b^
	>90%	20 (90.9)	11 (91.7)	
	<90%	2 (9.1)	1 (8.3)	
**Postoperative Complications**α				**0.721**^b^
	Yes/No	2/20 (9.1)	1/11 (8.3)	
	Fever (I)	–	1	
	Blood transfusion (II)	2	–	
Trifecta outcomes		22 (100)	12 (100)	
Pentafecta outcomes		20 (90.9)	11 (91.7)	0.941^a^

a Pearson's chi-square test,

b Fisher's exact test, CKD - chronic kidney disease, eGFR - estimated glomerular filtration rate,

α According to modified Clavien-Dindo classification,

* Continuous variables are presented as mean±SD, categorical variables as number (%)

## DISCUSSION

Previous studies reported that Off-C partial nephrectomy was associated with better preservation of RF but also higher estimated blood loss (7). However, a recent meta-analysis comparing Off-C and on-clamp robot assisted-LPN reported that Off-C group had shorter operation time and higher estimated blood loss. Oncological outcomes, overall complication, as well as early and late renal function were reported to be similar on smaller tumors (8). There is limited evidence in the literature on the superiority of laparoscopic Off-C versus on-clamp techniques. Hence, the data from CLOCK II study is awaited (9).

In the present study, it was found out that the renal functional preservation was better in R-LPN than T-LPN up to the 6th month after LPN. However, renal functional outcomes were found to be similar in both techniques on the postoperative first year. Furthermore, the mean relative change in renal function in the entire cohort was found to reduce by 3.9% when compared to the baseline eGFR at the end of the first year.

Preservation of RF to the extent possible and achieving satisfactory oncological outcomes is the main goals of LPN. Renal functional recovery after PN is reported to be influenced by a plenty of variables, including age, gender, preoperative RF, tumor size, WIT, the volume of the renal parenchyma preserved and concomitant comorbid diseases, as well. Ischemia time is reported to be a crucial modifiable risk factor that influences RF in patients who underwent PN in the short and long-term, postoperatively (10). Therefore, several PN techniques have been described to limit or even to eliminate the ischemia time including selective arterial clamping (11), early unclamping (12) and Off-C (13). Although the on-clamp technique that is commonly used in general practice during PN allows a bloodless field with enhanced visualization and facilitates tumor excision and renal reconstruction, it leads to ischemic injury on the renal parenchyma. In contrast, profuse bleeding during the Off-C technique may complicate precise identification of the surgical margin and calyceal entry, and renal parenchymal repair may also be challenging (14). In our study, EBL was found to be similar in both groups (170.23 vs. 150.83, p=0.790). We need to emphasize once again that the patients included in this study have low RENAL scores and the tumors were mostly exophytic. We speculate that the crucial point to achieve decreased blood loss during LPN is surgical experience. To gain the ability to complete intracorporeal suturing during renorrhaphy in a timely manner can be considered as a second important feature. On the other hand, the tumor excision technique could be argued. The surgeon is accustomed to use an aspirator on the non-dominant hand and laparoscopic scissors on the dominant hand during tumor excision. Thus, the view of the surgical field is getting better. In conclusion, surgical experience, modification of surgical technique according to the surgeon's preference, and tumors with lower RENAL scores are suitable to achieve lower blood loss during surgery.

The effect of Off-C LPN on RF has been evaluated by several comparative retrospective studies with limited number of patients. Off-C LPN provides an advantage for long-term preservation of RF in patients with solitary kidneys, while no difference was found between Off-C and on-clamp LPN in patients with contralateral functional kidney in terms of long-term RF (15).

In a comparative study in patients with solitary kidney, it has been reported that non-hilar clamping LPNs were more likely to have a <10% decrease in the long-term RF compared to clamping LPNs (16). However, it was stated that the patients who would benefit from Off-C LPN in patients with a contralateral functional kidney were those who had poorer preoperative RF. Except for this, the off-clamp technique had no advantage in terms of renal functional recovery in patients with normal kidney function (17).

In another study, functional and oncologic outcomes of 43 patients who underwent only Off-C T-LPN have been evaluated. This retrospective cohort study differs from our study in some aspects including the use of the PADUA scoring system to describe tumor complexity preoperatively (18), tumor excision and renorrhaphy technique used intraoperatively. However, in that study, the mean tumor size was 28.2mm, operation time was 172 min, and EBL was 341mL. Preoperative and 6-month postoperative mean eGFR values were 73 (range 37 to >90) and 71 (31 to >90) mL/min/1.73m2, respectively. The relative change in eGFR in month 6 was reported to be reduced by 2.8%. Positive surgical margin was identified in only one patient (19).

In a recent study, long-term (2-8 years) functional outcomes of on-clamp versus Off-C techniques have been compared in patients who underwent open PN for unilateral T1 and T2 renal tumor and had preoperative eGFR >60mL/min. After propensity score-matched analysis, the 472 Off-C and 157 on-clamp patients who underwent open PN were found to be similar in terms of age, gender, baseline eGFR, tumor size and comorbidities. In this study, it was concluded that the on-clamp technique had a higher probability of developing a stage ≥3b CKD compared to Off-C technique. The risk of developing CKD was also stated to be 7.3 fold higher in the on-clamp group during the follow-up (20).

There is no study available evaluating the effect of pure Off-C R-LPN on renal function in the literature. However, Porpiglia et al. have reported their initial experience of the mini-retroperitoneoscopic partial nephrectomy (mini-RPN) results of 10 patients having a low complex renal tumor (PADUA <8). In this case series, the mean tumor size, OT and EBL were 2.8 (range 1.5-5.5) cm, 91.5 min, 72mL, respectively. No intraoperative complications and no positive surgical margin were reported. Only one patient had a postoperative complication. The authors concluded that the initial report of Off-C mini-RPN has comparable outcomes compared to the conventional LPN. Nonetheless, preoperative and immediate early postoperative renal functional outcomes of mini-RPN have not been discussed in this report (21).

In the present study, although there were differences in terms of intraoperative variables when compared with previous studies in the literature, similar results were obtained in terms of tumor size, functional results, surgical margin negativity rates and postoperative complications (19-21).

It is also important to evaluate the effectiveness and the outcomes of minimally invasive nephron-sparing surgery in the short and long-term, using a common and standard definition. In this context, the concepts of trifecta and pentafecta, which are commonly used to evaluate the outcomes of LPN, are utilized. Although there are several different definitions for trifecta in the literature, the main goals of the trifecta outcomes in terms of LPN are achieving negative surgical margin, minimizing postoperative complications and WIT (22). Besides the definition of a diverse trifecta criterion, the characteristics of the patients and the tumor included in the studies, the surgical technique used and the surgical experience might have resulted in reporting disparate success rates (23, 24). Therefore, the trifecta outcomes indicated in the literature vary from 32% to 81% (25). In the present study, since neither hilar nor segmental vessels were clamped in any case, all LPN procedures had warm ischemia time of zero. The final pathology reported negative surgical margin in all patients included in this study. Although 4 patients required postoperative blood transfusion, none of the patients developed grade ≥3 complications of the modified Clavien-Dindo classification.

Recently, pentafecta criteria in minimally invasive PN are being used to evaluate the quality of the surgery. It is defined as trifecta criteria plus >90% preservation of eGFR and no stage upgrade of CKD up to 12 months postoperatively. In the literature, the papers that evaluated pentafecta outcomes for LPN are limited to robot-assisted LPN (RAPN). Stroup et al. have retrospectively compared the outcomes of 404 patients, who underwent 236 transperitoneal RAPN and 141 retroperitoneal RAPN and had similar demographic and clinical tumor characteristics, in terms of pentafecta and renal functional outcomes. The mean postoperative 6-month eGFR and ΔeGFR on the last follow-up were similar. They were 79.2 vs. 81.7mL/min/1.73m^2^, p=0.149 and 6.4 vs. 6.2mL/min/1.73m2, p=0.246, respectively. The achievement of pentafecta outcomes were reported as 33.9 vs. 43.3%, p=0.526 in T-RAPN vs. R-RAPN cohort. In our study, although GFR values showed statistically significant differences in T-LPN and R-LPN groups in the first six months, they were similar at the end of the first year. Similar findings were obtained in terms of the functional results evaluated during the last follow-up period. Nonetheless, in our study, the difference in eGFR from postoperative 1-day to 6-month suggests that it may be due to the surgical technique applied. It has been pointed out that pneumoperitoneum decreases blood flow and causes transient ischemia by compressing renal parenchyma and renal hilum. Nevertheless, this clinical effect was not clearly demonstrated when intraabdominal pressure was 12 to 15mmHg (26). We routinely prefer the carbon dioxide pressure that is increased up to 12mmHg to create a retroperitoneal or a transperitoneal space during LPN. Therefore, we consider that the ΔeGFR difference in the first 6 months may be affected by the pneumoperitoneum of the contralateral renal flow in the transperitoneal approach as well, even if the mean eGFR levels were found statistically significant.

There are some limitations to our study. The retrospective nature of the present study and the low number of patients in the groups are the main ones. However, we consider that it will be more accurate to evaluate a homogeneous cohort in terms of trifecta and pentafecta outcomes. Therefore, we excluded patients with a solitary kidney, unilateral multiple tumors, and bilateral tumors from the study. Moreover, the studies investigated the outcomes of Off-C LPN in the literature also have limited number of patients as well. Previous studies have evaluated the safety and effectiveness of the technique; there are no long-term follow-up results available. This paper is a study evaluating the initial experience of a single surgeon in terms of both surgical and functional outcomes of Off-C LPN. On the other hand, renal scintigraphy might be useful to compare the percentages of both kidney functions between each other instead of GFR measurement that represent the two kidneys. However, our study design was retrospective and we do not routinely apply renal scintigraphy before and after PN in clinical practice. Therefore, a prospective study with scintigraphy may be helpful to achieve a more accurate renal functional evaluation.

## CONCLUSIONS

According to the present study, transperitoneal and retroperitoneal off-C LPN techniques were found to have similar outcomes in terms of preservation of renal function at the end of the first year postoperatively. Off-C LPN may be considered as a safe and effective treatment option with high rates of trifecta and pentafecta outcomes in selected patients having non-complex renal tumors.

## ABBREVIATIONS

BMI: = body mass index
CAD: = coronary artery disease
CKD-EPI: = Chronic Kidney Disease Epidemiology Collaboration
CT: = computed tomography
DM: = diabetes mellitus
EBL: = estimated blood loss
eGFR: = estimated glomerular filtration rate
Hgb: = hemoglobin
HT: = hypertension
LPN: = laparoscopic partial nephrectomy
Off-C: = Off-clamp
OT: = operation time
PN: = partial nephrectomy
RF: = renal function
RN: = radical nephrectomy
RNS: = R.E.N.A.L nephrometry score
WIT: = warm ischemia time

## References

[1] 1. Ljungberg B, Albiges L, Abu-Ghanem Y, Bensalah K, Dabestani S, Fernández-Pello S, et al. European Association of Urology Guidelines on Renal Cell Carcinoma: The 2019 Update. Eur Urol. 2019;75:799-810. 10.1016/j.eururo.2019.02.011 30803729

[2] 2. Volpe A, Blute ML, Ficarra V, Gill IS, Kutikov A, Porpiglia F, et al. Renal Ischemia and Function After Partial Nephrectomy: A Collaborative Review of the Literature. Eur Urol. 2015;68:61-74. 10.1016/j.eururo.2015.01.025 25703575

[3] 3. Kutikov A, Uzzo RG. The R.E.N.A.L. nephrometry score: a comprehensive standardized system for quantitating renal tumor size, location and depth. J Urol. 2009;182:844-53. 10.1016/j.juro.2009.05.035 19616235

[4] 4. Levey AS, Stevens LA. Estimating GFR using the CKD Epidemiology Collaboration (CKD-EPI) creatinine equation: more accurate GFR estimates, lower CKD prevalence estimates, and better risk predictions. Am J Kidney Dis. 2010;55:622-7. 10.1053/j.ajkd.2010.02.337 PMC2846308 20338463

[5] 5. Eckardt KU, Berns JS, Rocco MV, Kasiske BL. Definition and classification of CKD: the debate should be about patient prognosis--a position statement from KDOQI and KDIGO. Am J Kidney Dis. 2009;53:915-20. 10.1053/j.ajkd.2009.04.001 19406541

[6] 6. Dindo D, Demartines N, Clavien PA. Classification of surgical complications: a new proposal with evaluation in a cohort of 6336 patients and results of a survey. Ann Surg. 2004;240:205-13. 10.1097/01.sla.0000133083.54934.ae PMC1360123 15273542

[7] 7. Hotston MR, Keeley FX. Laparoscopic partial nephrectomy without ischemia. Arch Esp Urol. 2013;66:146-51. 23406810

[8] 8. Antonelli A, Veccia A, Francavilla S, Bertolo R, Bove P, Hampton LJ, et al. On-clamp versus off-clamp robotic partial nephrectomy: A systematic review and meta-analysis. Urologia. 2019;86:52-62. 10.1177/0391560319847847 31179885

[9] 9. Bove P, Bertolo R, Sandri M, Farullo G, Cipriani C, Leonardo C, et al. Assessing the impact of renal artery clamping during laparoscopic partial nephrectomy (LPN) for small renal masses: the rationale and design of the CLamp vs Off Clamp Kidney during LPN (CLOCK II) randomised phase III trial. BJU Int. 2019;124:365-7. 10.1111/bju.14779 31009140

[10] 10. Klatte T, Ficarra V, Gratzke C, Kaouk J, Kutikov A, Macchi V, et al. A Literature Review of Renal Surgical Anatomy and Surgical Strategies for Partial Nephrectomy. Eur Urol. 2015;68:980-92. 10.1016/j.eururo.2015.04.010 PMC4994971 25911061

[11] 11. Yezdani M, Yu SJ, Lee DI. Selective Arterial Clamping Versus Hilar Clamping for Minimally Invasive Partial Nephrectomy. Curr Urol Rep. 2016;17:40. 10.1007/s11934-016-0596-0 26968420

[12] 12. Lah K, Desai D, Chabert C, Gericke C, Gianduzzo T. Early vascular unclamping reduces warm ischaemia time in robot-assisted laparoscopic partial nephrectomy. F1000Res. 2015;4:108. 10.12688/f1000research.6276.1 PMC4431378 26069733

[13] 13. Boyarsky L, Stein A, Konstantinovsky A, Goldin D, Klein I, May T, et al. Retrospective Analysis of Laparoscopic Partial Nephrectomies Using the Zero Ischemia Technique. Urol Int. 2017;99:257-61. 10.1159/000454989 28259881

[14] 14. Klatte T, Ficarra V, Gratzke C, Kaouk J, Kutikov A, Macchi V, et al. A Literature Review of Renal Surgical Anatomy and Surgical Strategies for Partial Nephrectomy. Eur Urol. 2015;68:980-92. 10.1016/j.eururo.2015.04.010 PMC4994971 25911061

[15] 15. Marconi L, Desai MM, Ficarra V, Porpiglia F, Van Poppel H. Renal Preservation and Partial Nephrectomy: Patient and Surgical Factors. Eur Urol Focus. 2016;2:589-600. 10.1016/j.euf.2017.02.012 28723490

[16] 16. Wszolek MF, Kenney PA, Lee Y, Libertino JA. Comparison of hilar clamping and non-hilar clamping partial nephrectomy for tumours involving a solitary kidney. BJU Int. 2011;107:1886-92. 10.1111/j.1464-410X.2010.09713.x 21070570

[17] 17. Porpiglia F, Bertolo R, Amparore D, Podio V, Angusti T, Veltri A, et al. Evaluation of functional outcomes after laparoscopic partial nephrectomy using renal scintigraphy: clamped vs clampless technique. BJU Int. 2015;115:606-12 10.1111/bju.12834 24913695

[18] 18. Ficarra V, Novara G, Secco S, Macchi V, Porzionato A, De Caro R, et al. Preoperative aspects and dimensions used for an anatomical (PADUA) classification of renal tumours in patients who are candidates for nephron-sparing surgery. Eur Urol. 2009;56:786-93. 10.1016/j.eururo.2009.07.040 19665284

[19] 19. Browne C, Lonergan PE, Bolton EM, D’Arcy F, Lynch TH. A single centre experience of zero-ischaemia laparoscopic partial nephrectomy in Ireland. Ir J Med Sci. 2017;186:1023-6. 10.1007/s11845-017-1562-7 28124281

[20] 20. Simone G, Capitanio U, Tuderti G, Presicce F, Leonardo C, Ferriero M, et al. On-clamp versus off-clamp partial nephrectomy: Propensity score-matched comparison of long-term functional outcomes. Int J Urol. 2019;26:985-91. 10.1111/iju.14079 31342589

[21] 21. Porpiglia F, Bertolo R, Amparore D, Cattaneo G, Fiori C. Mini-retroperitoneoscopic clampless partial nephrectomy for “low-complexity” renal tumours (PADUA score ≤8). Eur Urol. 2014;66:778-83. 10.1016/j.eururo.2014.06.001 24954790

[22] 22. Mehra K, Manikandan R, Dorairajan LN, Sreerag S, Jain A, Bokka SH. Trifecta Outcomes in Open, Laparoscopy or Robotic Partial Nephrectomy: Does the Surgical Approach Matter? J Kidney Cancer VHL. 2019;6:8-12. 10.15586/jkcvhl.2019.115 PMC6532825 31149561

[23] 23. Yerram NK, Dagenais J, Bryk DJ, Nandanan N, Maurice MJ, Mouracade P, et al. Trifecta Outcomes in Multifocal Tumors: A Comparison Between Robotic and Open Partial Nephrectomy. J Endourol. 2018;32:615-620. 10.1089/end.2018.0134 29790375

[24] 24. Bindayi A, Autorino R, Capitanio U, Pavan N, Mir MC, Antonelli A, et al. Trifecta Outcomes of Partial Nephrectomy in Patients Over 75 Years Old: Analysis of the REnal SURGery in Elderly (RESURGE) Group. Eur Urol Focus. 2020;6:982-90. 10.1016/j.euf.2019.02.010 30799289

[25] 25. Demirdag C, Citgez S, Gevher F, Simsekoglu F, Yalcin V. Trifecta Outcomes of Laparoscopic Partial Nephrectomy for T1a and T1b Renal Tumors: A Single-Center Experience in a Tertiary Care Institution. J Laparoendosc Adv Surg Tech A. 2019;29:790-5. 10.1089/lap.2018.0756 30724656

[26] 26. Adamy A, Favaretto RL, Nogueira L, Savage C, Russo P, Coleman J, et al. Recovery of renal function after open and laparoscopic partial nephrectomy. Eur Urol. 2010;58:596-601. 10.1016/j.eururo.2010.05.044 20554375

